# Supplementary material for: Coiled-coil structure of meiosis protein TEX12 and conformational regulation by its C-terminal tip
Source: Commun Biol. 2022 Sep 7;5:921. doi: 10.1038/s42003-022-03886-9 (PMC9452514; doi:10.1038/s42003-022-03886-9)
Supplement: Supplementary file 2 — Supplementary Information [file 42003_2022_3886_MOESM2_ESM.pdf]

Supplementary Information

Coiled-coil structure of meiosis protein TEX12 and conformational regulation by its C-terminal tip

James M. Dunce, Lucy J. Salmon and Owen R. Davies

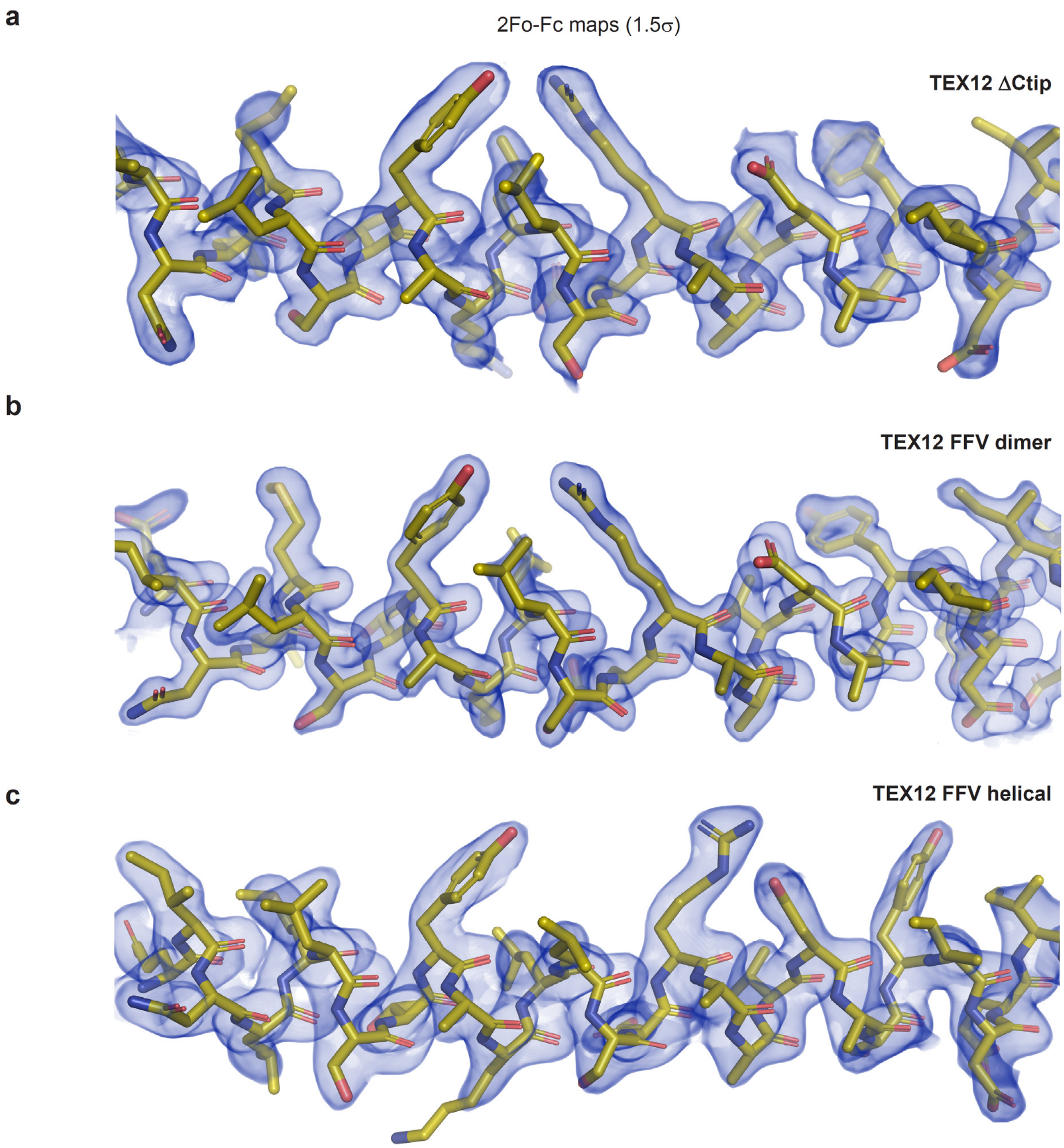

Supplementary Figure 1

Crystal structures of TEX12 core DCtip and FFV mutants.

(**a-c**) 2Fo-Fc electron density maps (1.5σ) of TEX12 (**a**) ΔCtip, (**b**) FFV in a dimeric conformation and (**c**) FFV in a dimeric helical conformation, superimposed on their refined crystallographic models.

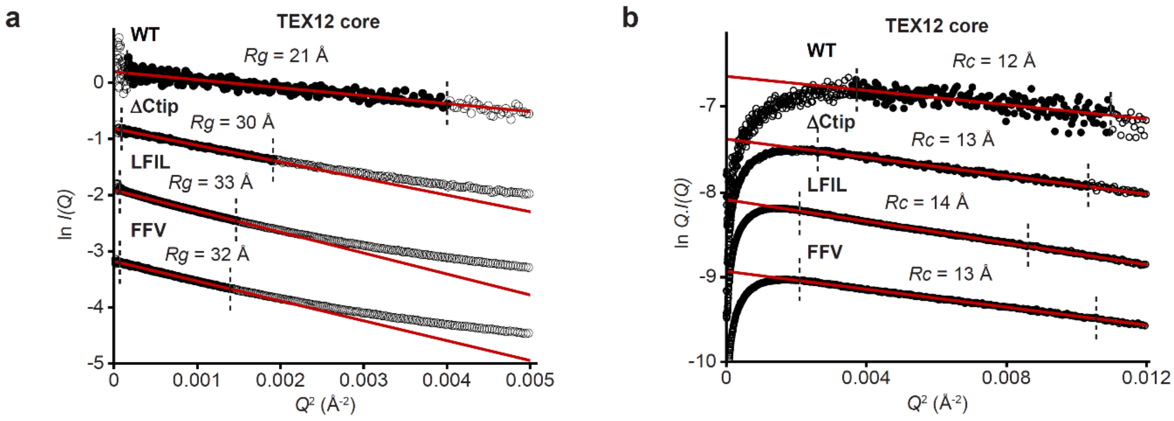

Supplementary Figure 2

SEC-SAXS analysis of TEX12 Ctip mutants.

(**a,b**) SEC-SAXS analysis of TEX12 wild-type, ΔCtip, LFIL and FFV. (**a**) SAXS Guinier analysis to determine the radius of gyration (*Rg*); linear fits are shown in red, with the fitted data range highlighted in black and demarcated by dashed lines. The *Q.Rg* values were < 1.3 and *Rg* was calculated as 21 Å, 30 Å, 33 Å and 32 Å, respectively. (**b**) SAXS Guinier analysis to determine the radius of gyration of the cross-section (*Rc*); linear fits are shown in red, with the fitted data range highlighted in black and demarcated by dashed lines. The *Q.Rc* values were < 1.3 and *Rc* was calculated as 12 Å, 13 Å, 14 Å and 13 Å, respectively.

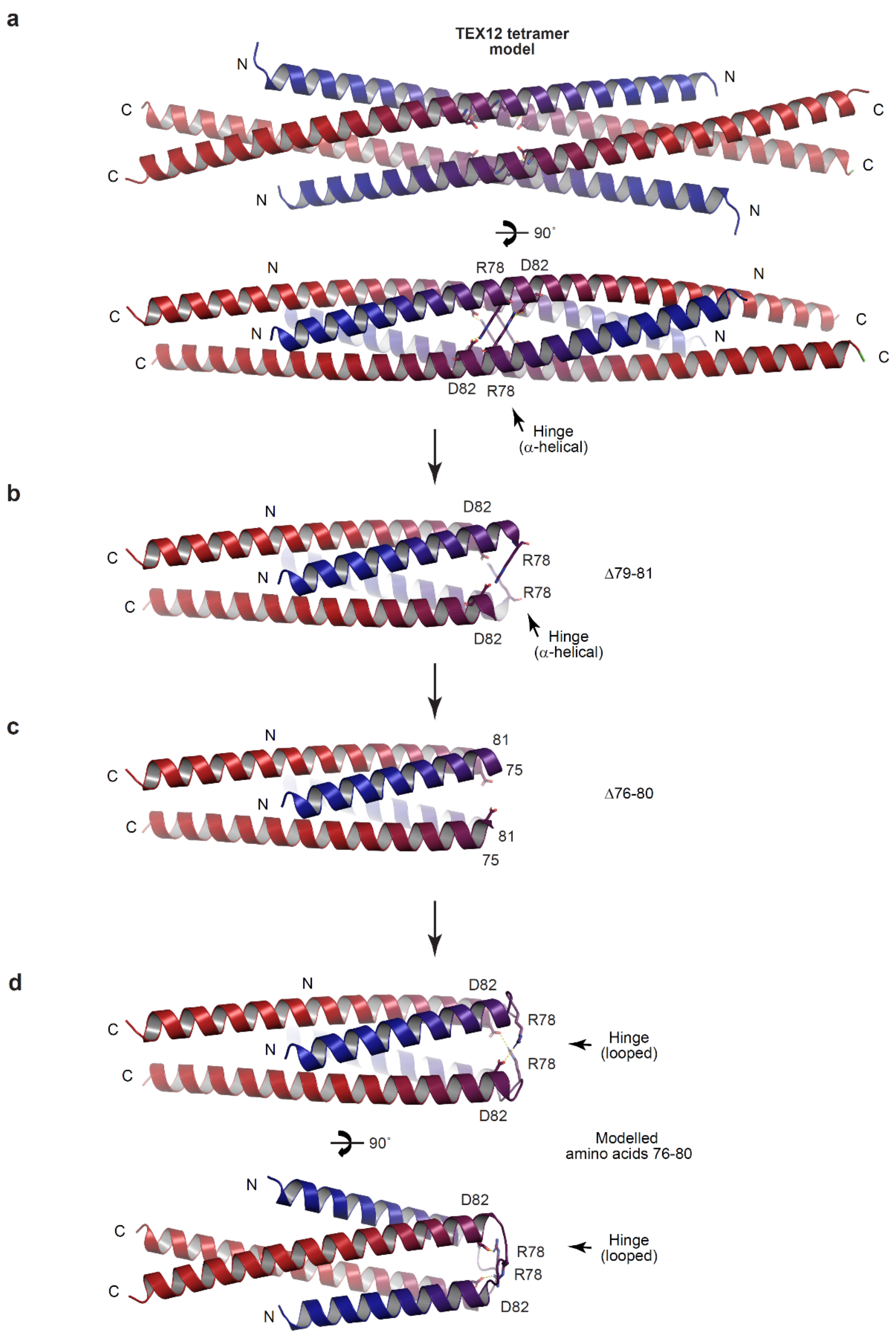

Supplementary Figure 3

Modelling the TEX12 compact dimer structure.

(**a**) The FFV tetramer structure contains the four R78-D82 salt bridges (constituting the α-helical hinges) at its centre of symmetry. (**b**) One half of the FFV tetramer was taken, retaining one hinge of

two R78-D82 salt bridges, and additionally removing amino-acids 79-81. (**c**) On the basis of the overhang of N-terminal relative to C-termini helices, and the need for both ends to be proximally located, the deletion was extended to amino-acids 76-80, leaving closely associated amino-acids 75 and 81 at the helical ends. (**d**) The missing amino-acids 76-80 were modelled as loops between helical ends, resulting in the TEX12 compact dimer model. In this structure, R78-D82 salt bridges are re-established, with R78 and D82 residues located within the looped hinge and C-terminal helices, respectively.

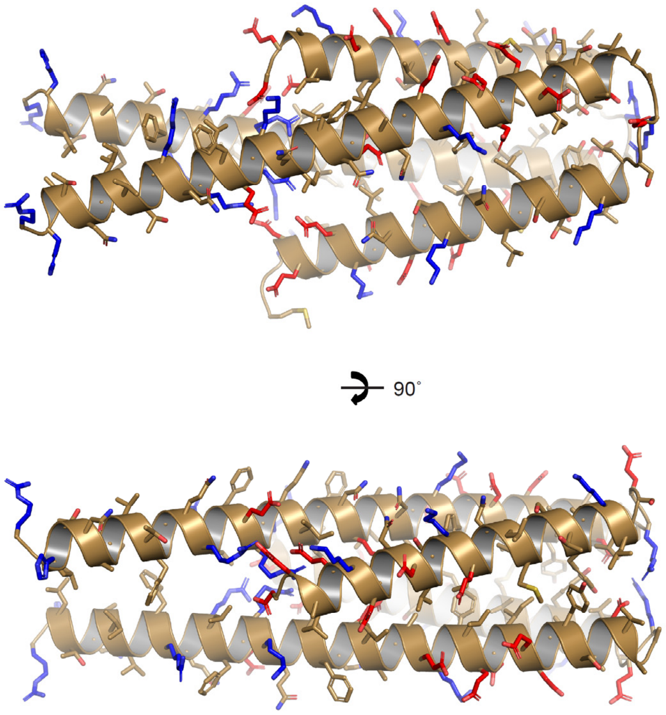

Supplementary Figure 4

Amino-acid composition of the TEX12 compact dimer.

TEX12 compact dimer model with amino-acid side chains coloured as wheat (hydrophobic), red (acidic) and blue (basic); uncharged polar side chains are coloured in wheat with red (oxygen) and blue (nitrogen) functional groups.

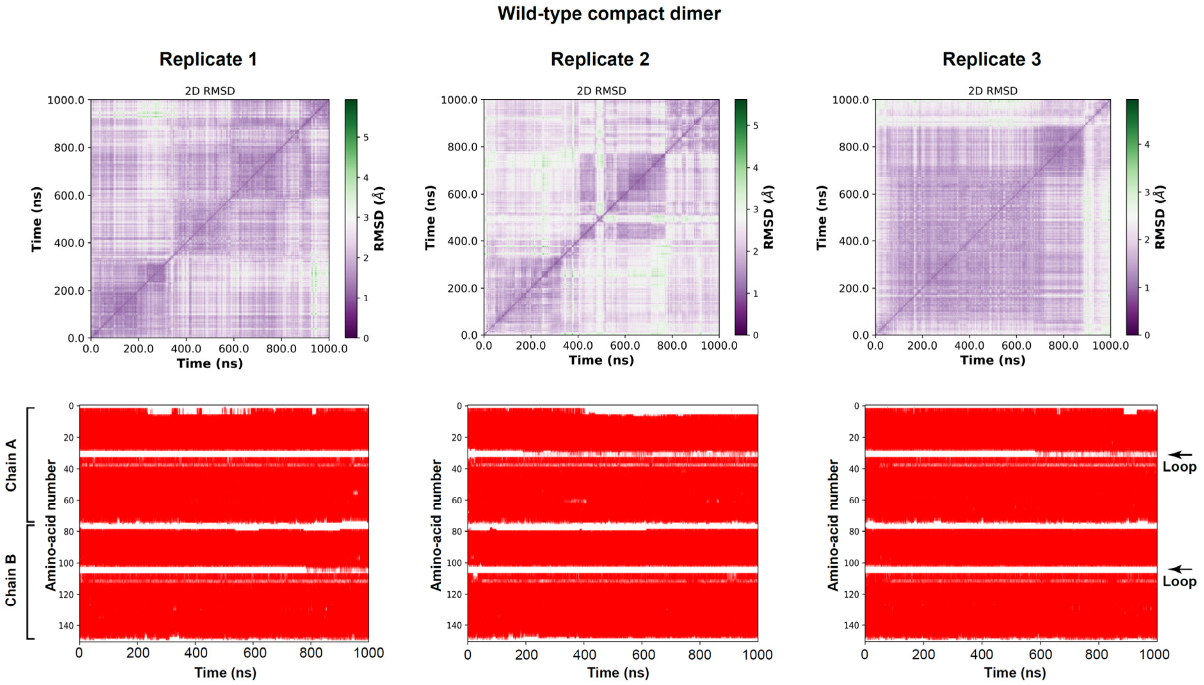

Supplementary Figure 5

Molecular dynamics simulations of the TEX12 compact dimer.

2D r.m.s. deviation plots (top) and secondary structure composition per amino-acid with α-helical structure coloured in red (bottom) for the three replicates of 1-us molecular dynamics simulations of the TEX12 compact dimer structure shown in Figure 7.

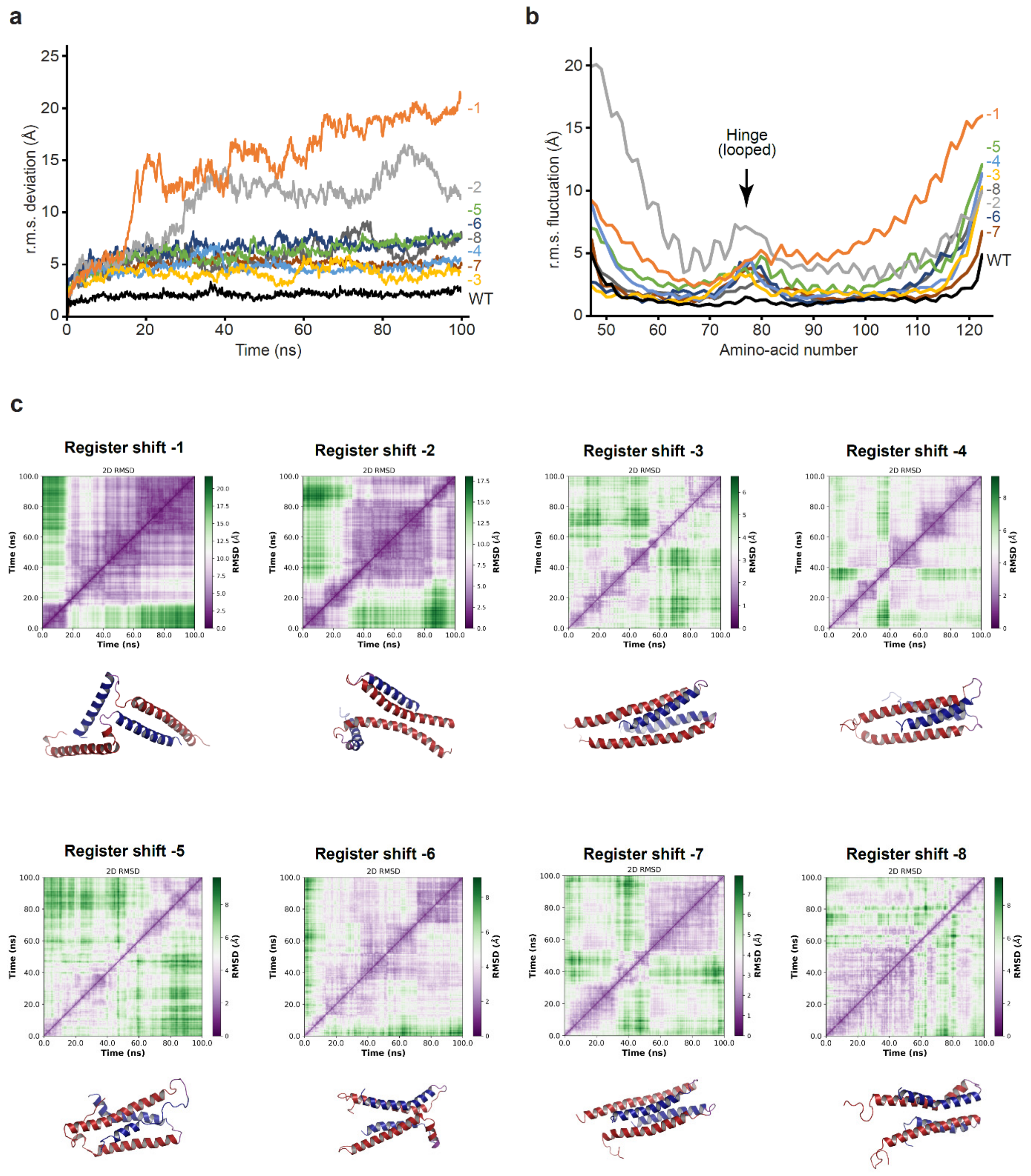

Supplementary Figure 6

Molecular dynamics simulations of the TEX12 compact dimer harbouring sequence register shifts.

(**a-c**) Analysis of the TEX12 compact dimer structure, upon introduction of sequence register shifts of between 1-8 amino-acids (labelled as -1 to -8), across 100-ns molecular dynamics simulations performed at 37°C. (**a**) Overall r.m.s. deviations and (**b**) individual amino-acid r.m.s. fluctuations; data

from a representative wild-type (WT) simulation are shown in black for comparison. (**c**) 2D r.m.s. deviations shown alongside the resultant structures from the register shift 100-ns simulations.

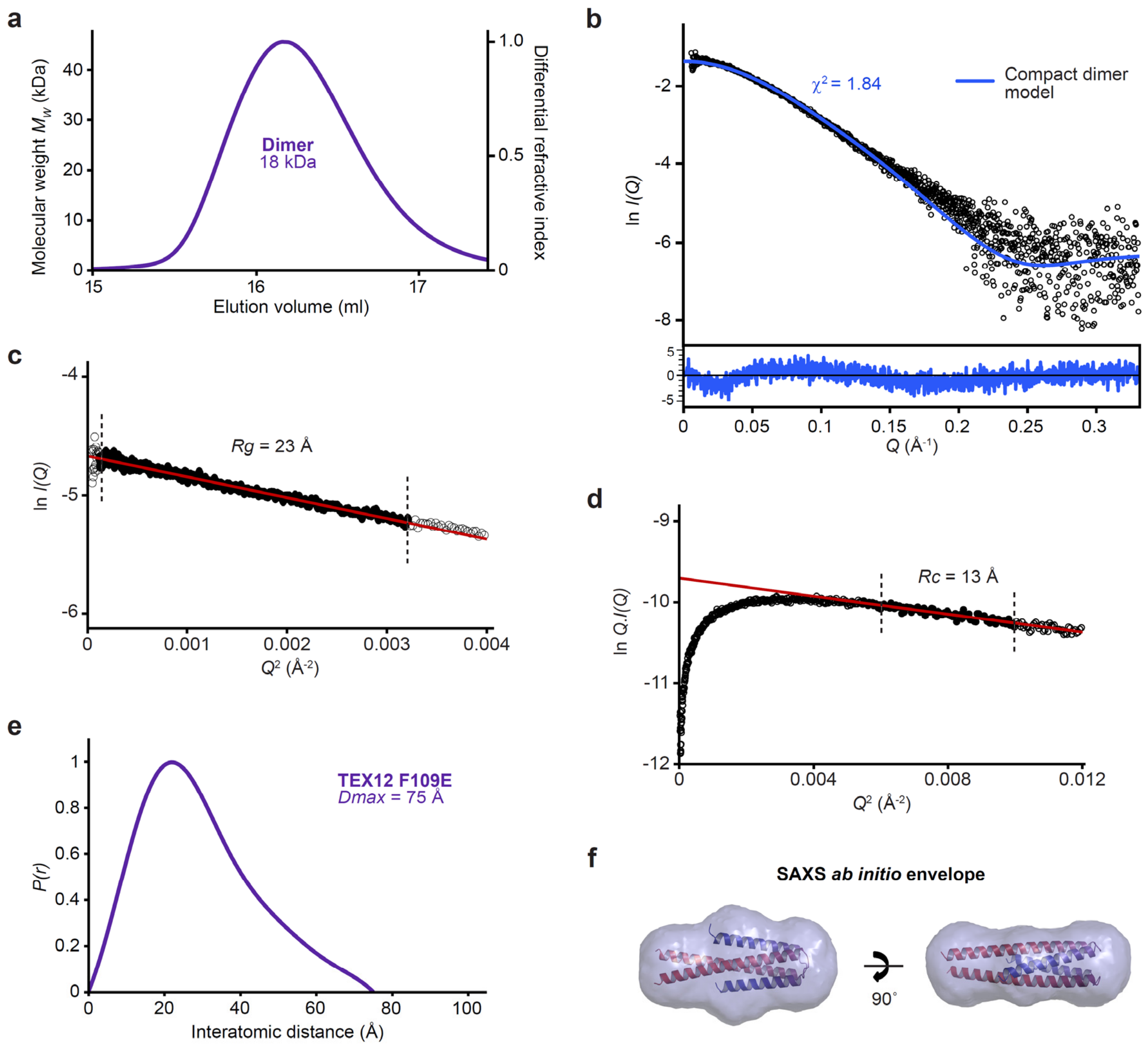

Supplementary Figure 7

TEX12 is stabilised in a dimeric conformation by F109E mutation.

(**a**) SEC-MALS analysis demonstrating that F109E forms an 18 kDa dimer (theoretical - 18 kDa). (**b-f**) SEC-SAXS analysis of TEX12 F109E. (**b**) SAXS scattering data overlaid with the theoretical scattering curve of the compact dimer model; the χ^2 value is indicated and residuals are shown for the fit (inset). (**c**) SAXS Guinier analysis to determine the radius of gyration (*Rg*); linear fits are shown in red, with the fitted data range highlighted in black and demarcated by dashed lines. The *Q.Rg* values were < 1.3 and *Rg* was calculated as 23 Å. (**d**) SAXS Guinier analysis to determine the radius of gyration of the cross-section (*Rc*); linear fits are shown in red, with the fitted data range highlighted in black and demarcated by dashed lines. The *Q.Rc* values were < 1.3 and *Rc* was calculated as 13 Å. (**e**) SAXS *P(r)* interatomic

distance distributions in which maximum dimension (*Dmax*) is indicated. (**g**) SAXS *ab initio* model of TEX12 F109E. A filtered averaged model from 30 independent *DAMMIF* runs is shown with the TEX12 compact dimer model docked into the SAXS envelopes.

a

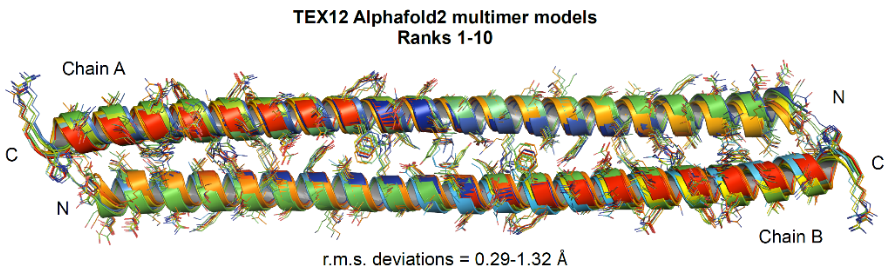

b

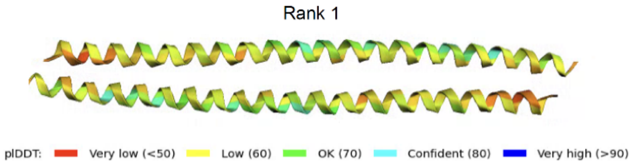

c

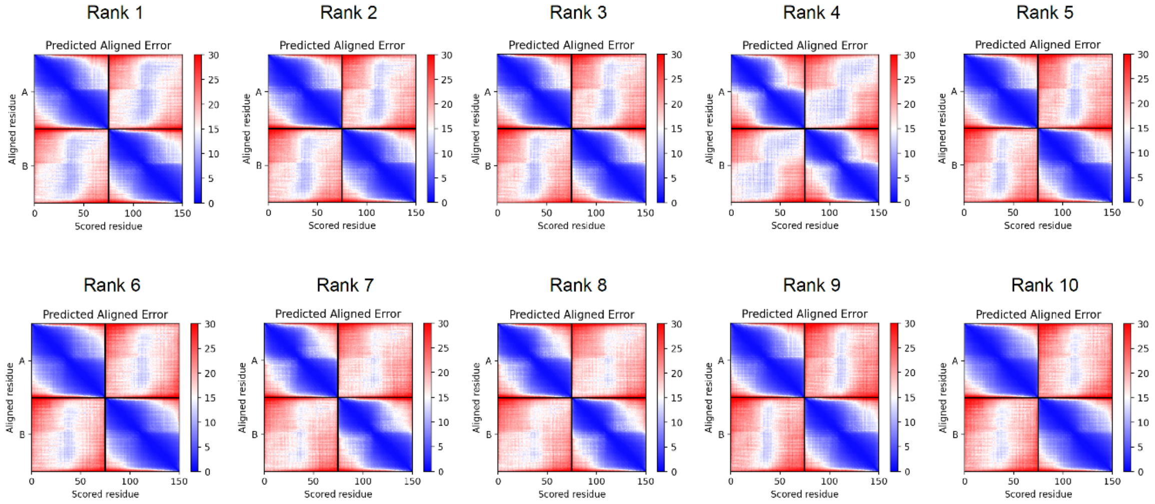

d

	r.m.s. deviation (Å)				
	TEX12 ΔCtip AA	TEX12 ΔCtip AB	TEX12 FFV dimer	TEX12 FFV helical	TEX12 compact dimer
**Rank 1 model**	10.32	9.64	9.88	2.43	31.71

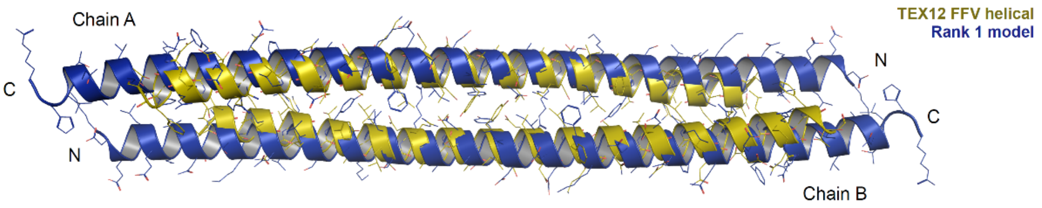

Supplementary Figure 8

Alphafold2 multimer models of the TEX12 dimer.

(**a**) Superposition of the top ten ranked models (out of 25) generated by Alphafold2 multimer, showing r.m.s. deviations of between 0.29-1.33 Å. (**b**) Top ranked model coloured by pLDDT value,

ranging from red (very low confidence) to blue (very high confidence). (**c**) Paired alignment errors of the top ten ranked models coloured from blue (low error) to red (high error). (**d**) R.m.s. deviations of the top ranked model to the four dimeric structures presented herein and the TEX12 compact dimer model (top), and a superposition of the top ranked model (blue) and the TEX12 FFV helical dimer (yellow).

Supplementary Table 1 Summary of SEC-SAXS data

TEX12 core	WT	ΔCtip	LFIL	FFV	F109E
**SASBDB accession**	SASDNN5	SASDNP5	SASDNQ5	SASDNR5	SASDNS5
**Guinier analysis**					
I(0) (cm^{-1})	0.0036 ±2.8x10^{-5}	0.037 ±6.6x10^{-5}	0.028 ±2.7x10^{-5}	0.068 ±4.9x10^{-5}	0.0042 ±1.1x10^{-5}
Rg (Å)	21 ±0.28	30 ±0.08	33 ±0.04	32 ±0.04	23 ±0.10
Rc (Å)	11.6	12.7	13.8	12.6	12.9
q_{min} ($Å^{-1}$)	0.013	0.0085	0.0021	0.0075	0.011
***P(r)* analysis**					
I(0) (cm^{-1})	0.0036 ±2.1x10^{-5}	0.038 ±6.2x10^{-5}	0.029 ±2.6x10^{-5}	0.068 ±4.9x10^{-5}	0.0041 ±9.3x10^{-6}
Rg (Å)	21 ±0.12	31 ±0.08	36 ±0.05	34 ±0.04	23 ±0.07
D_{max} (Å)	66	110	129	120	75
Porod volume ($Å^3$)	32545	52968	67181	58031	36598
MW from Porod volume (kDa)	19	31	39	34	22
V_C ($Å^2$)	210	326	383	360	239
MW from V_C (kDa)	17	29	36	32	20
DAMMIF ab initio* modelling *(30 models)					
Symmetry	P1	N/A	N/A	P2	P2
NSD mean and s.d.	0.675 ±0.033	N/A	N/A	0.833 ±0.056	0.567 ±0.062
χ^2 (reference model)	1.04	N/A	N/A	1.20	1.24
**Structural modelling**					
CRYSOL (χ^2)	1.14	1.54	N/A	1.94	1.84
