# Supplementary material for: Coiled-coil structure of meiosis protein TEX12 and conformational regulation by its C-terminal tip
Source: Commun Biol. 2022 Sep 7;5:921. doi: 10.1038/s42003-022-03886-9 (PMC9452514; doi:10.1038/s42003-022-03886-9)
Supplement: Supplementary file 3 — Description of Additional Supplementary Files [file 42003_2022_3886_MOESM3_ESM.pdf]

Description of Additional Supplementary Files

File name: Supplementary Data 1
Description: The source data behind the MD analyses shown in the paper.
